# Genome wide transcriptome analysis provides bases on colonic mucosal immune system development affected by colostrum feeding strategies in neonatal calves

**DOI:** 10.1186/s12864-018-5017-y

**Published:** 2018-08-28

**Authors:** Zhixiong He, Amanda Fischer, Yang Song, Michael Steele, Le Luo Guan

**Affiliations:** 1 Key Laboratory of Agro-ecological Processes in Subtropical Region, Institute of Subtropical Agriculture, Chinese Academy of Sciences, Changsha, Hunan 410125 People’s Republic of China; 2 Department of Agriculture, Food and Nutritional Sciences, University of Alberta, Edmonton, T6G 2P5 Canada

**Keywords:** Calf, Colon, Colostrum feeding, RNA-sequencing

## Abstract

**Background:**

Delivery of colostrum within the first several hours after birth is vital for establishing successful passive immunity in neonatal dairy calves. However, it is unclear whether a difference in colostrum feeding strategy can affect the development of the calf gastrointestinal tract. The aim of this study was to evaluate the effect of colostrum feeding time within the first 12 h after birth on the colonic mucosal immune system in neonatal calves using a genome wide transcriptome analysis.

**Results:**

RNA sequencing-based transcriptome analysis of colon tissues collected from 27 male Holstein calves which were randomly assigned to one of three colostrum feeding strategies – (immediately after birth (TRT0); 6 h after birth (TRT6); 12 h after birth (TRT12)) – and euthanized at 51 h of age detected 15,935 ± 210, 15,332 ± 415, and 15,539 ± 440 expressed genes in the colon under three treatments, respectively. The core transcriptome of the colon included 12,678 genes, with enriched “cellular process” and “metabolic process” as the top two biological functions with 802 of them being immune function related genes. Principal component analysis of the colon transcriptomes did not display a clear separation by colostrum feeding strategy and differential abundance analyses showed no significant difference in the expression of immune related genes among the treatments. Additionally, a weighted gene co-expression network analysis identified 4 significant (|correlation| > 0.50 and *p* ≤ 0.05) gene modules consisting of 122 immune related genes, which were positively or negatively correlated with the abundance of *Lactobacillus* and *Faecalibacterium prausnitzii* in the colon.

**Conclusion:**

Transcriptome analysis indicates that the development of the colonic mucosal immune system in neonatal calves may be independent of the timing of initial colostrum meal within 12 h after birth. Our results also provide a molecular understanding of colonic biological function in neonatal calves and extends knowledge on how host gene expression profiles are associated with the abundance of specific bacterial groups in the colon.

**Electronic supplementary material:**

The online version of this article (10.1186/s12864-018-5017-y) contains supplementary material, which is available to authorized users.

## Background

Due to the presence of syndesmochorial placenta in dairy cows, immunoglobulins (Igs) cannot be transferred from the dam to the fetus, which results in the newborn calf being agammaglobulinemic [1]. Ingestion and absorption of adequate amounts of colostral IgG, known as the passive transfer of immunity, is a prerequisite for neonatal calf health and survival [2–4]. For instance, inadequate IgG absorption from colostrum is associated with higher risks of enteric infections caused by *rotavirus* and *Cryptosporidium spp.* [5]. It has been suggested that the timely delivery of high quality and quantity colostrum within the first 24 h after birth is vital for the establishment of passive immunity in calves [6, 7]. Recent studies have shown that substantially larger IgG intakes are required when feeding a colostrum meal more than 2 h after birth in order to achieve successful passive transfer [8], suggesting the importance of feeding colostrum to newborn calves immediately after birth. In addition to containing IgG, colostrum is also rich in a variety of bioactive components, such as growth factors, hormones and prebiotics [9], which are important for cellular growth and differentiation, and influence gut function, metabolism and immune reactions [10, 11]. Under practical management, not all calves can be fed within a few hours after birth. Therefore, we hypothesized that delaying colostrum feeding, even within the first hours after birth, would result in a delayed effect on the development of the mucosal immune system in the gastrointestinal tract.

In pre-ruminants, the dietary substrates that are not digested in the stomach and small intestine reach the colon and bacterial fermentation of nutrient substrates can produce short chain fatty acids (SCFAs). It has been reported that the SCFAs including butyrate and propionate play important roles in regulation gut physiology, barrier function, immune system and inflammatory response [12–14], suggesting that the colon functions as a site of immune response and the microbes in the colon could directly or indirectly affect the immune function of the colon via microbial production of SCFAs. However, knowledge on the functional development of the colon in pre-ruminants is limited and the functional development of the colon in neonatal calves has not been investigated. The objective of this study was to characterize the function of colon tissues during early life using RNA-sequencing (RNA-seq) based transcriptome analysis and to evaluate whether colonic mucosal immune system development of neonatal calves can be affected by a delayed colostrum feeding.

## Methods

### Animal, feeding and sample collection

The Livestock Care Committee of the University of Alberta reviewed and approved all procedures for the animal trial (AUP00001595), which complied with the guidelines of the Canadian Council of Animal Care [15]. Calves were born at the Dairy Research and Technology Centre of the University of Alberta from February to September of 2016, and only the male calves from a singleton birth with a body weight between 35 and 55 kg were chosen for the experiment. As a result, 27 male Holstein calves, 12 from primiparous heifers (H) and 15 from multiparous cows (C) with an average birth weight of 42.6 ± 4.21 kg (mean ± SD), were used in the current study. Immediately after birth, the calves were removed from the dam (without physical contact), ear tagged, weighed and housed in individual pens with straw bedding. The calves were randomly assigned to one of 3 feeding strategies for the initial colostrum meal after birth (7.5% of birth body weight; *n* = 9 each group, 4 from H and 5 from C): 1) fed immediately after birth (TRT0); 2) fed at 6 h after birth (TRT6); or 3) fed at 12 h after birth (TRT12). Pooled and pasteurized colostrum (62 g/L of IgG; Saskatoon Colostrum Company Ltd.; Saskatoon, SK, Canada) was thawed from frozen in a water bath and heated to 39 °C prior to feeding. At their respective feeding time, colostrum was tubed to calves using an esophageal tube feeder. Twelve hours after the colostrum meal, all calves were fed a milk replacer diet (260 g/kg crude protein, 180 g/kg crude fat; Excel Pro-Gro Calf Milk Replacer, Grober Nutrition, Cambridge, Ontario, Canada) at a volume of 2.5% of birth body weight every 6 h of life. There was no difference in calf birth weight among colostrum treatments and the sample collection was performed following the procedures described in the study of Fischer et al. [16]. Briefly, the calves were euthanized with an intravenous injection of pentobarbital sodium at 51 h of age, and 5-cm segments of the colon were collected distal to the ileo-cecal junction during dissection. The samples, rinsed three times with PBS buffer to remove ingesta, were immediately frozen in liquid-N_2_ and stored at − 80 °C freezer.

### RNA extraction and sequencing

Total RNA was extracted from frozen colon tissues using a mirVana total RNA Isolation Kit (Ambion, Carlsbad, CA, USA) following the manufacturer’s instruction. Firstly, ground frozen tissue (~ 100 mg) was mixed thoroughly with 10 volumes of mirVana Lysis/Binding Solution by vortexing for 30 s. Samples were then homogenized by a Precellys 24 homogenizer (Bertin Technologies, Montigny, France) for 30 s twice. After homogenization, an equal volume of Acid-Phenol:Chloroform (Ambion) was added, followed by vortexing the mixture for 1 min and centrifugation for 5 min at 10,000×g. The resulting aqueous solution was mixed thoroughly with 1.25 volumes of 100% ethanol and filtered through a mirVana column in a 700 μl aliquot twice. After the column was washed 3 times using the given wash solution, the RNA remaining in the column were eluted in nuclease-free water at 95 °C. The quality and quantity of RNA were measured by an Agilent 2200 TapeStation (Agilent Technologies, Santa Clara, CA, USA) and a Qubit 2.0 Fluorometer (Invitrogen, Carlsbad, CA, USA), respectively. All RNA samples had least a 1.8 ratio of A260nm/A280nm measured by Nanodrop (Nanodrop Technologies, Wilmington, DE, USA) and had an integrity number of 7.0 or higher. All RNA samples were then stored at − 80 °C.

Total RNA (100 ng) from each sample was used for library construction using TruSeq RNA Library Prep Kit v2 (Illumina, San Diego, CA, USA) according to the manufacturer’s instructions. Firstly, total RNA was fragmented and the first and second strand cDNA were synthesized through reverse transcription. The cDNA was then subjected to end repair and 3′-end adenylation, followed by ligation of index adapters and PCR enrichment (98 °C for 30 s, followed by 15 cycles of: 98 °C for 10 s, 60 °C for 30 s, 72 °C for 30 s, and 72 °C for 5 min; the final products were held at 4 °C). Libraries (250–270 bp) were verified using an Agilent 2200 TapeStation (Agilent Technologies) and quantified with a Qubit fluorometer using a Qubit dsDNA HS Assay Kit (Invitrogen). The individual indexed libraries were then pooled and sequenced at Génome Québec (Montréal, Canada) on the Illumina HiSeq 4000 platform (Illumina) to obtain 100 bp paired-end reads.

### RNA sequencing data processing and normalization

Sequencing reads that did not pass the Illumina chastity filter in CASAVA 1.8 (Illumina) were discarded and filtered reads were then aligned to the reference bovine genome (UMD 3.1) using Tophat 2.1.1 [17, 18]. The BAM alignment files generated from Tophat were sorted using Samtools (v1.4) [19] and converted to SAM files. The SAM files were analyzed using HTSeq-count to obtain gene read counts based on the annotation from ENSEMBL bovine genes [20]. Reads that were assigned to more than one gene were not counted by HTSeq-count. To compare the expression profiles, the read counts of each gene were normalized to counts per million (CPM) using the following formula: CPM = (gene read counts/total mapped counts per library) × 1,000,000. Principal Component Analysis (PCA) was conducted with cluster samples based on gene expression data using R (R version 3.4.2).

### Transcriptome profiles and differential expression analysis

Genes with at least one CPM in all calves were considered as core genes. The functions of the core genes in the colon were annotated using protein annotation through evolutionary relationship (PANTHER; Version 12.0, released July 10, 2017) gene list analysis tool [21]. Functional classification of genes in categories such as molecular function, biological process and cellular component, were based on gene ontology (GO) annotations.

Differentially expressed (DE) genes were analyzed using the bioinformatics package edgeR (v3.5) [22]. The sequencing data was firstly filtered by keeping genes with at least one CPM in 50% of the samples per treatment group (5 out of 9 animals in each group). To investigate the overall colostrum treatment and parity effect on colonic transcriptome profiles, 4 contrasts including TRT6 vs. TRT0, TRT12 vs. TRT0, TRT12 vs. TRT6, and C vs. H were conducted. Another 9 contrasts including TRT6_H vs. TRT0_H, TRT12_H vs. TRT0_H, TRT12_H vs. TRT6_H, TRT6_C vs. TRT0_C, TRT12_C vs. TRT0_C, TRT12_C vs. TRT6_C, TRT0_C vs. TRT0_H, TRT6_C vs. TRT6_H, and TRT12_C vs. TRT12_H were also conducted to identify transcriptomic differences within colostrum treatment and parity. *p* values were multiple-test corrected for false discovery rate (FDR) using the Benjamini and Hochberg method, and significant DE genes were declared at fold change ≥1.2 and FDR ≤ 0.10.

### Analysis of specific genes related to mucosal immune system

To investigate the colonic mucosal immune system development affected by colostrum treatment and parity, the sequencing data of immune related genes was further analyzed. The immune related gene list was obtained from ImmPort database [23] based on their categorized function in cytokine, cytokine receptor, interleukin (IL), interleukin receptor, interferon, interferon receptor, tumor necrosis factor (TNF) family member, TNF family member receptor, transforming growth factor (TGF-b) family member, TGF-b family member receptor, chemokine, chemokine receptor, T cell receptor (TCR) signaling pathway, breakpoint cluster region (BCR) signaling pathway, natural killer cell, antigen processing and presentation, or antimicrobial (shown in Additional file 1). In addition, *IL10*, *signal transducers and activators of transcription 3* (*STAT3*), *nuclear factor kappa B 1* (*NFKB1*), *tumor necrosis factor* (*TNF*), *toll-like receptors* (*TLR*) *2*, *TLR4*, *claudin 4* (*CLDN4*) and *peptidoglycan recognition protein 1* (*PGLYRP1*) genes, which have been previously investigated in dairy calves, were also included as the representative genes of intestinal mucosal immunity [24–27].

### Gene co-expression network analysis

To identify the modules and key genes associated with bacterial populations in the colon, a gene co-expression network analysis was conducted based on their relationship with the copy numbers of *Lactobacillus*, *Faecalibacterium prausnitzii* (*F. prausnitzii*) and *Bifidobacterium* associated with the colon tissue and content using Weighted Gene Co-expression Network Analysis (WGCNA; v1.49) [28]. The microbial data were obtained from Fischer et al. [16] who used the same colon samples and reported above bacterial abundances using quantitative PCR. Automatic network construction and module detection function (blockwiseModules within the WGCNA) were used to generate subsets of genes, and the modules were correlated with abundance of *Lactobacillus*, *F. prausnitzii* and *Bifidobacterium* in the colon based on the following major parameters: minModuleSize of 30, reassign Threshold of 0, and mergeCutHeight of 0.30. The resulting gene modules were assigned with different color names by the software and Module-Trait relationships were calculated by Pearson correlation. Modules with significant correlations (|correlation| > 0.50 and *P* ≤ 0.05) were kept and further investigated.

## Results

### Colonic transcriptome profiles of neonatal dairy calves

**Fig. 1:**
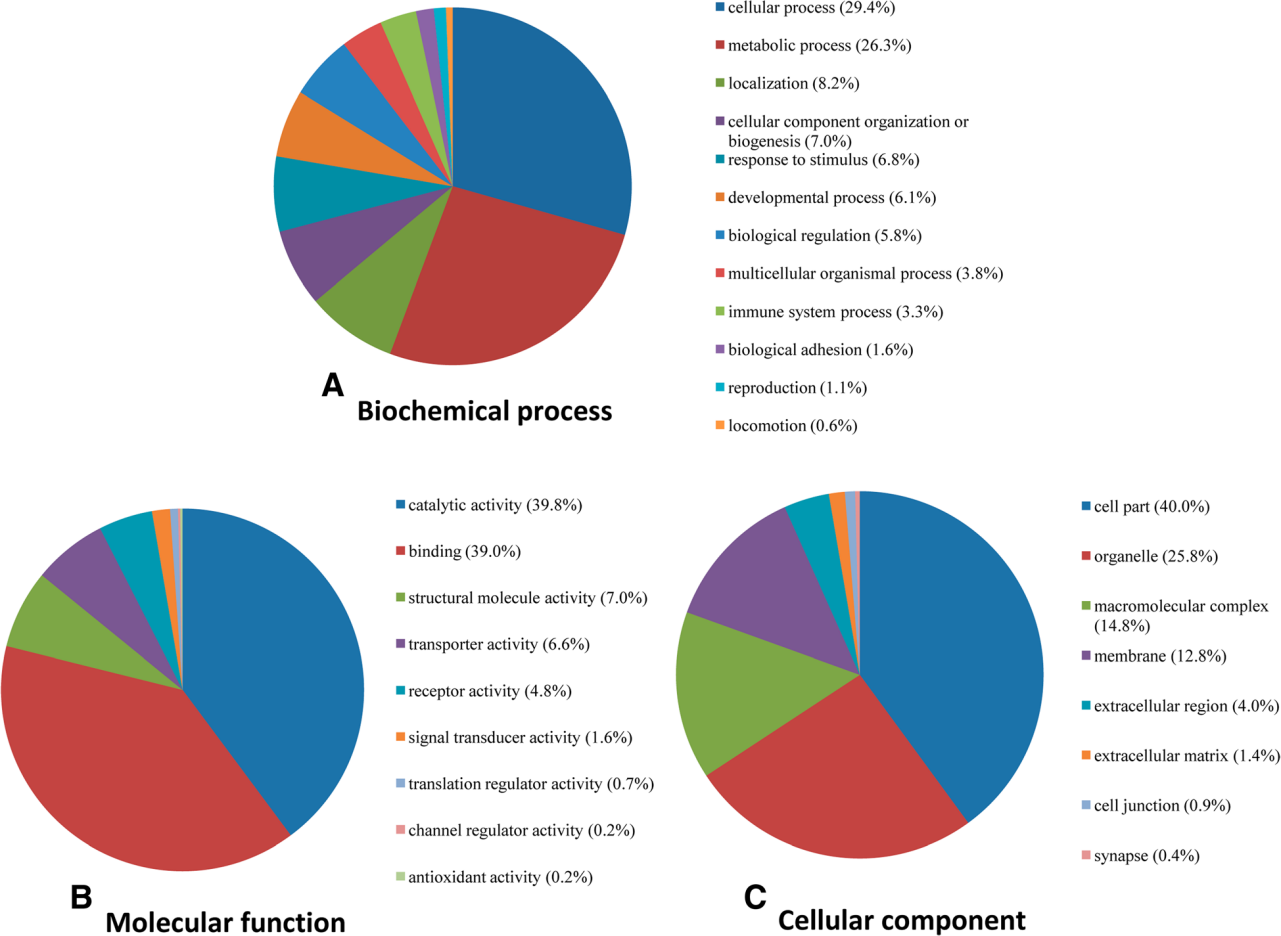
Functional classification of the core genes in the colon of dairy calves as identified by PANTHER. The percentage of core genes involved in (**a**) Biological processes, (**b**) Molecular functions, and (**c**) cellular components

A total of 877 million paired-end reads were obtained from all 27 samples, with an average of 32.5 ± 9.1 million reads per sample (Additional file 2). The overall mapped rate to the bovine reference genome (UMD 3.1) was 87.4 ± 6.2%. The total number of genes prior to any data filtering across the 27 samples was 24,617 with 12,678 core genes (genes with at least one CPM in all the samples) identified. From these core genes, 11,613 genes could be annotated by PANTHER and functional analysis of the core genes showed that the top biochemical processes (Fig. 1a) were “cellular process” (29.4% of genes) and “metabolic process” (26.3% of genes); the top molecular functions (Fig. 1b) were “catalytic activity” (39.8% of genes) and “binding” (39.0% of genes); the top cellular components (Fig. 1c) were “cell part” (40.0% of genes) and “organelle” (25.8% of genes). The symbol and CPM of genes used for core functional analysis are listed in Additional file 3.

### Colonic transcriptome changes in response to delayed colostrum feeding in neonatal dairy calves

**Fig. 2:**
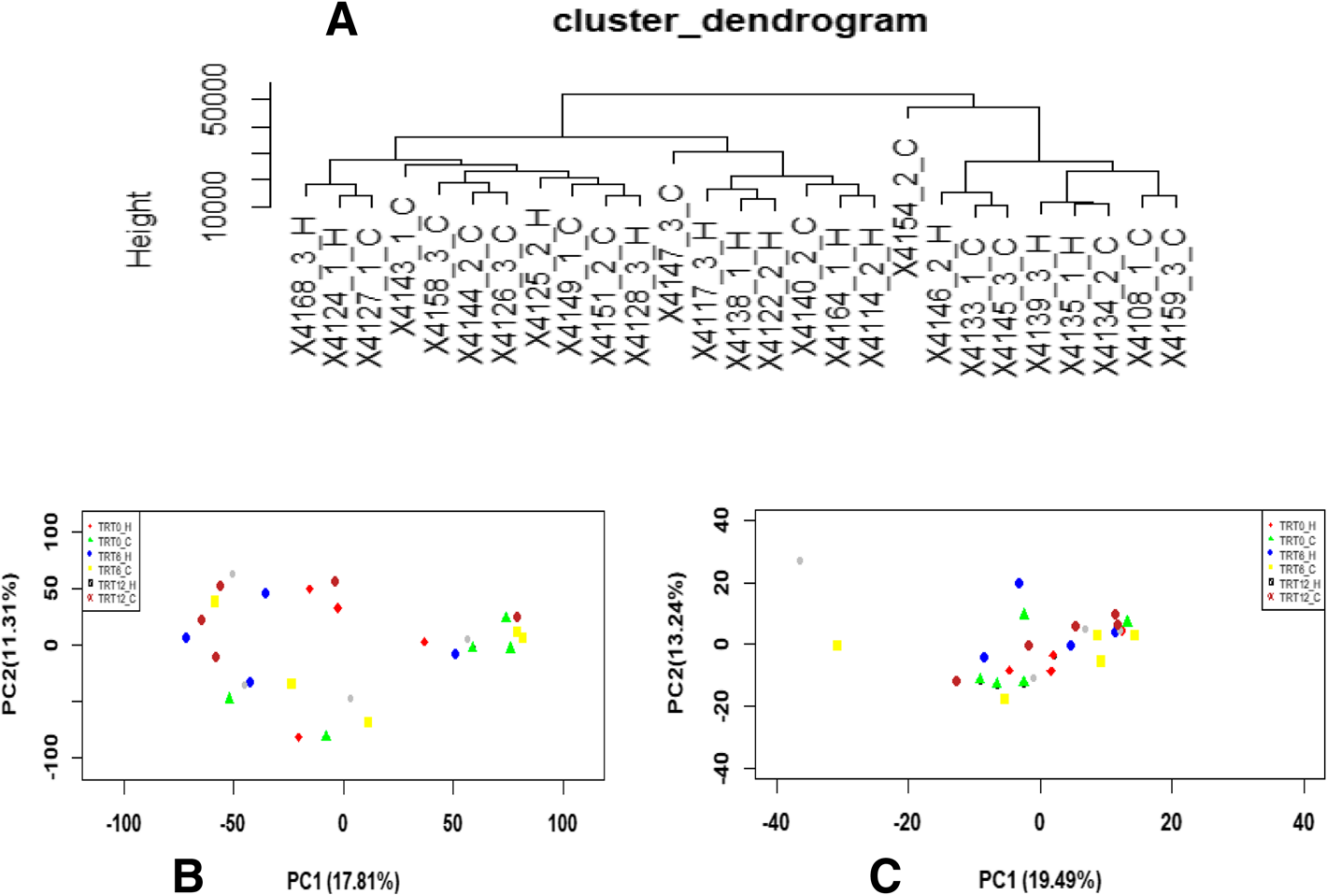
Cluster and PCA plot of transcriptome profiles. **a** Hierarchical cluster of transcriptomes. ID represents one sample from animal. For instance, “×4168_3_H” means the sample from animal No. ×4168 which was assigned to the third group (TRT12) and from primiparous heifers (H). **b** PCA plot of transcriptomes for the colon samples from TRT0_H (red color), TRT0_C (green color), TRT6_H (blue color), TRT6_C (yellow color), TRT12_H (grey color) and TRT12_C (brown color). The X and Y-axis represent the first two principle components. The percentage value in the bracket represents the percentage of variance explained by that principle component. **c** PCA plot of the immune-related genes

**Fig. 3:**
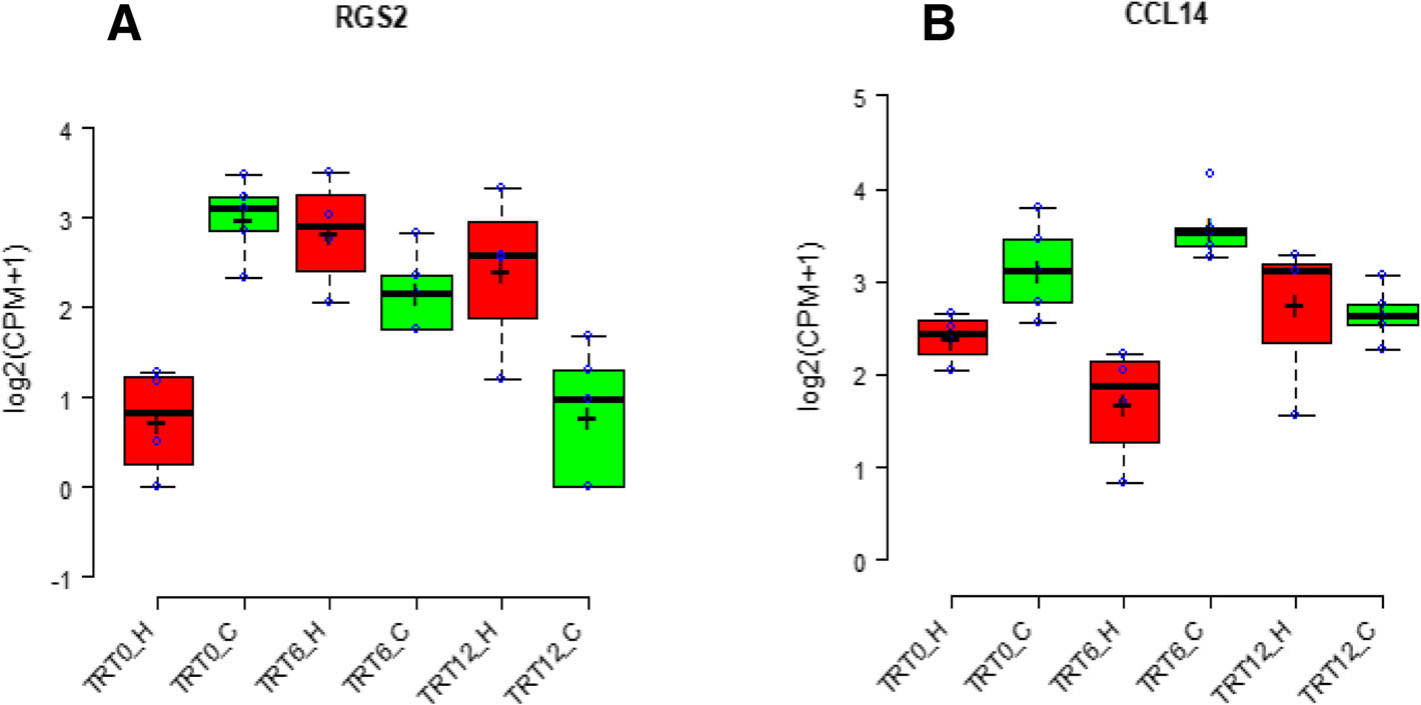
The expression level of RGS2 (**a**) and CCL14 (**b**) in dairy calves with varying colostrum feeding time

The number of expressed genes in the colon of TRT0, TRT6 and TRT12 calves was 15,935 ± 210, 15,332 ± 415, and 15,539 ± 440, respectively. The transcriptome profiles did not reveal a clustering pattern either by colostrum treatment or parity (Fig. 2a). Principal component analysis of the transcriptome profiles also did not display a clear separation either by colostrum treatment or parity (Fig. 2b). Differential gene expression analyses across/within colostrum treatment and parity showed that almost none of the genes passed a FDR correction for multiple testing, with the exception that *regulator of G protein signaling 2 (RGS2)* was up-regulated (fold change = 8.04, FDR = 0.032) in the colon of TRT6_H calves when compared to that of TRT0_H, down-regulated (fold change = 6.74, FDR = 0.002) in the colon of TRT12_C calves when compared to that of TRT0_C, and up-regulated (fold change = 8.25, FDR = 0.006) in the colon of TRT0_C calves when compared to that of TRT0_H. In addition, the expression of *Chemokine (C-C motif) ligand 14 (CCL14)* was up-regulated (fold change = 4.26, FDR = 0.029) in the colon of TRT6_C when compared to that in TRT6_H (Table 1, Fig. 3).

**Table 1:** Differentially expressed genes affected by colostrum treatment and dam parity

	DE gene	Log (FC)	FDR
Within parity			
TRT6_H vs. TRT0_H	*RGS2*	3.01	0.032
TRT12_H vs. TRT0_H	NS	NS	NS
TRT12_H vs. TRT6_H	NS	NS	NS
TRT6_C vs. TRT0_C	NS	NS	NS
TRT12_C vs. TRT0_C	*RGS2*	−2.75	0.002
TRT12_C vs. TRT6_C	NS	NS	NS
Within treatment			
TRT0_C vs. TRT0_H	*RGS2*	3.04	0.006
TRT6_C vs. TRT6_H	*CCL14*	2.09	0.029
TRT12_C vs. TRT12_H	NS	NS	NS
Across parity			
TRT6 vs. TRT0	NS	NS	NS
TRT12 vs. TRT0	NS	NS	NS
TRT12 vs. TRT6	NS	NS	NS
Across treatment			
C vs. H	NS	NS	NS

Note: H = primiparous heifers; C = multiparous cows; *RGS2* = *regulator of G-protein signaling 2*; NS = not significant; *CCL14* = *chemokine (C-C motif) ligand 14*; FC = fold change; cutoff for DE genes was FDR < 0.10, and |FC| > 1.2

### Assessment of the expression of genes involved in immune function

**Fig. 4:**
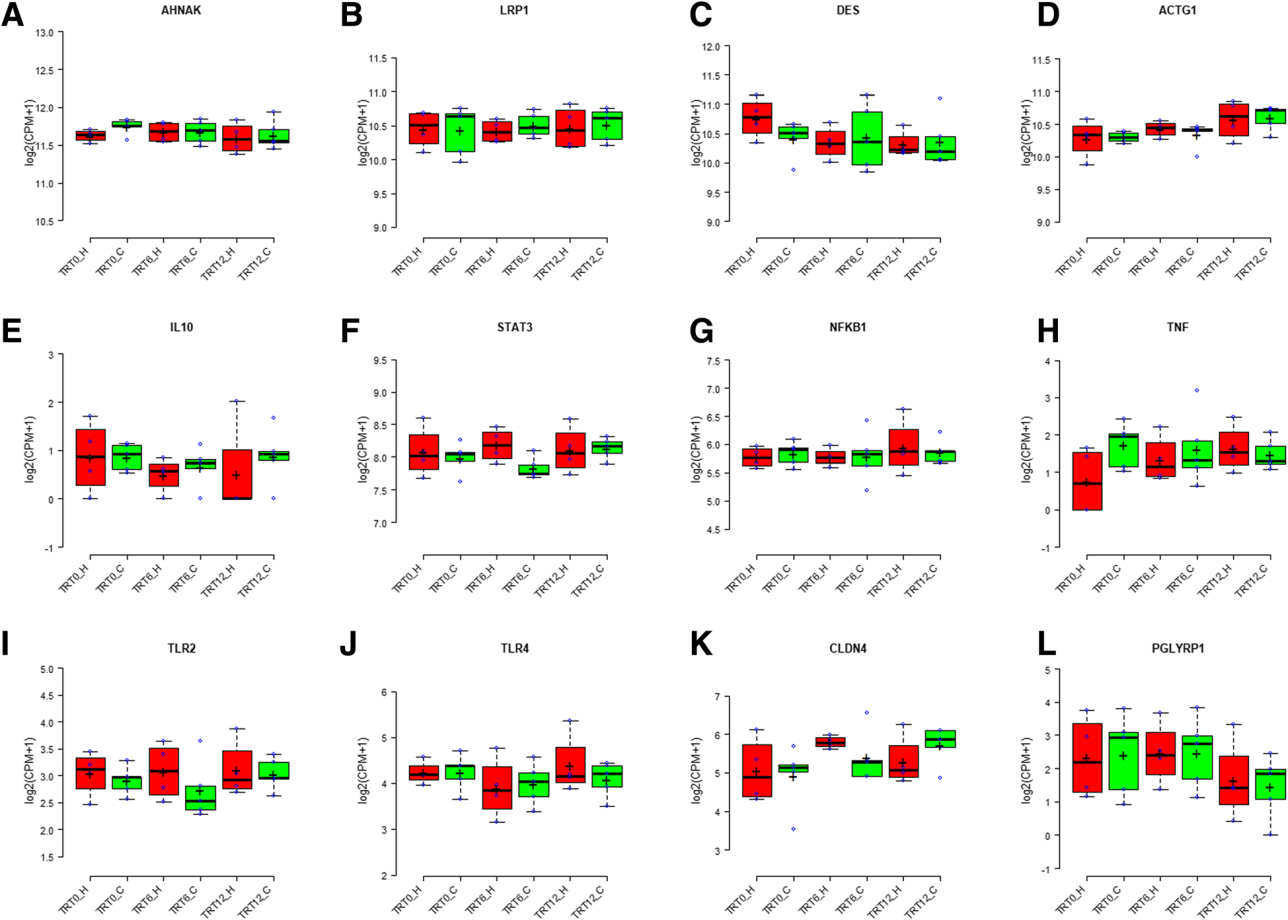
The expression level of *AHNAK* (**a**), *LRP1* (**b**), *DES* (**c**), *ACTG1* (**d**), *IL10* (**e**), *STAT3* (**f**), *NFKB1* (**g**), *TNF* (**h**), *TLR2* (**i**), *TLR4* (**j**), *CLDN4* (**k**) and *PGLYRP1* (**l**) in dairy calves with varying colostrum feeding time

A total of 802 genes were identified to be related to immune function and were subjected to further analysis (Additional file 1). Similar to the whole transcriptome, the expression of these immune related genes did not display a clear separation either by colostrum treatment or parity (Fig. 2c). The top 10 expressed immune related genes, based on an averaged CPM value, were *AHNAK nucleoprotein (AHNAK)*, *LDL receptor related protein (LRP1)*, desmin *(DES)*, *actin gamma 1 (ACTG1)*, *heat shock protein family A member 8 (HSPA8)*, *solute carrier family 40 member 1* (*SLC40A1)*, *alpha-2-macroglobulin (A2M)*, *calnexin (CANX)*, *calreticulin (CALR)* and *tropomyosin 2 (TPM2)*, and the top four expressed immune related genes were all categorized as antimicrobials (Table 2). The expression profiles of these genes are plotted in Fig. 4 and their mean, SD, min, max values of CPM are presented in Table 2.

**Table 2:** Expression summary statistics (difference between individual real CPM and the average CPM) of the representative immune related genes

	Mean	SD	Min	Max	Range	Rank	Category
*AHNAK*	3252	327	2655	3927	1271	1	Antimicrobials
*LRP1*	1434	234	995	1803	808	2	Antimicrobials
*DES*	1433	394	923	2285	1362	3	Antimicrobials
*ACTG1*	1385	217	935	1843	908	4	Antimicrobials
*IL10*	0.8	0.8	0.0	3.0	3.0	780	Antimicrobials, Cytokines, TCR signaling Pathway
*STAT3*	268	51	196	385	189	26	Antimicrobials
*NFKB1*	57	12	35	97	62	153	Antimicrobials, BCR Signaling Pathway, TCR signaling Pathway
*TNF*	2.1	1.7	0.0	8.1	8.1	686	Antimicrobials, Cytokines, Natural Killer Cell
*TLR2*	7.2	2.5	3.9	13.7	9.8	525	Antimicrobials
*TLR4*	17.5	6.5	7.8	40.3	32.5	389	Antimicrobials
*CLDN4*	44	19	10	92	82.3	210	Antimicrobials
*PGLYRP1*	4.7	4.2	0.0	13.2	13.2	586	Antimicrobials

Note: CPM = counts per million

### Exploration of modules and key genes associated with bacterial colonization in the colon of neonatal dairy calves

To investigate if there was a relationship between the expression of genes and the bacterial populations in the colon, a network-based analysis WGCNA was further conducted. A total of 15,578 genes remained for the input of WGCNA. After setting a minimum module size of 30 and merging modules with highly correlated eigengenes, a total of 23 modules were found. All modules were assigned a color and depicted in the hierarchical clustering dendrogram outlined in Additional file 4. Among the 23 co-expressed gene modules, 1 and 3 modules were significantly (|correlation| > 0.50 and *P* ≤ 0.05) correlated with the abundance of *Lactobacillus* and *F. prausnitzii* in the colon, respectively (Table 3, Additional file 5). For *Lactobacillus*, the Greenyellow module (264 genes including 11 immune related genes) had a negative correlation (*r* = − 0.52 and *p* = 0.006) with its abundance in the colon tissue. For *F. prausnitzii*, the Red module (863 genes including 46 immune related genes) had a positive correlation (*r* = 0.60 and *p* = 0.001) with its abundance in the colon content, and the Lightgreen module (62 genes including 2 immune related genes) and the Grey module (1505 genes including 63 immune related genes) had a negative correlation (*r* = − 0.82 and *p* < 0.001 for the Lightgreen module; *r* = − 0.68 and *p* < 0.001 for the Grey module) with its abundance in the tissue. There was no significant correlation detected between the modules and *Bifidobacterium* abundance in the colon. The gene list and name within each module significantly declared are available in Additional file 6.

**Table 3:** Gene modules correlated with the bacterial abundance in the colon identified using a weighted gene co-expression network analysis

Module	Gene number (number of immune genes)	lac_C_P	lac_T_P	fea_C_P	fea_T_P	bif_C_P	bif_T_P
Black	488	NS	NS	NS	NS	NS	NS
Blue	2332	NS	NS	NS	NS	NS	NS
Brown	2102	NS	NS	NS	NS	NS	NS
Cyan	140	NS	NS	NS	NS	NS	NS
Darkgreen	60	NS	NS	NS	NS	NS	NS
Darkred	60	NS	NS	NS	NS	NS	NS
Green	955	NS	NS	NS	NS	NS	NS
Greenyellow	264 (11)	NS	−0.52 (*p* = 0.006)	NS	NS	NS	NS
Grey	1505 (63)	NS	NS	NS	−0.68 (*p* = 9.00E-05)	NS	NS
Grey60	79	NS	NS	NS	NS	NS	NS
Lightcyan	88	NS	NS	NS	NS	NS	NS
Lightgreen	62 (2)	NS	NS	−0.82 (*p* = 1.00–07)	NS	NS	NS
Lightyellow	62	NS	NS	NS	NS	NS	NS
Magenta	367	NS	NS	NS	NS	NS	NS
Midnightblue	93	NS	NS	NS	NS	NS	NS
Pink	435	NS	NS	NS	NS	NS	NS
Purple	351	NS	NS	NS	NS	NS	NS
Red	863 (46)	NS	NS	0.60 (*p* = 0.001)	NS	NS	NS
Royalblue	62	NS	NS	NS	NS	NS	NS
Salmon	189	NS	NS	NS	NS	NS	NS
Tan	204	NS	NS	NS	NS	NS	NS
Turquoise	3730	NS	NS	NS	NS	NS	NS
Yellow	1086	NS	NS	NS	NS	NS	NS

Note: Only the number of immune genes in significantly correlated modules were shown in the brackets; NS = not significant; lac_C_*p* = *Lactobacillus* population from the colon content; lac_T_*p* = *Lactobacillus* population from the attached colon; fea_C_*p* = *Faecali bacterium prausnitzii* population from the colon content; fea_T_*p* = *Faecali bacterium prausnitzii* population from the attached colon; bif_C_*p = Bifidobacterium* population from the colon content; bif_T_*p = Bifidobacterium* population from the attached colon

## Discussion

To our knowledge, this is the first study reporting the transcriptome profiles of the colon and their changes in response to colostrum feeding strategies in neonatal calves. In ruminants, the function of the colon has been overlooked in the past; however, a recent study revealed that fermentation in the colon could provide energy to the host through microbial production of SCFAs in pre-weaned calves [14], suggesting that the colon plays an important role in nutrient metabolism. In the present study, transcriptome analysis revealed that the top biochemical processes of the colon in neonatal calves are involved in cellular and metabolic processes, suggesting that the colon plays an important role in nutrient metabolism in neonatal calves – a finding in line with other animal models which highlight the role of the production of SCFAs by the colonic microbiota to maintain energy homeostasis [29]. Interestingly, gene expression during the maturation of the colon in rats has been shown to be up- or down-regulated by an antibiotic treatment from day 7 of life until day 17 or 21, and their functions were related to ion transport processes [30] – an outcome which was not observed in the neonatal calves in the present study. This may suggest the presence of age-related functional development and maturation of the colon in neonates, which was not observed in the present study due to the colonic transcriptome profiles being analyzed only at 51 h of life, and not any further.

The gastrointestinal tract acts as the first defense against pathogenic infections by providing physical barriers and activating innate and adaptive immune responses [31], which is particularly important in agammaglobulinemic calves in order to prevent infection and sickness, such as diarrhea. Song et al. [14] suggested that the colon of dairy calves harvest an abundance of diverse microbes soon after birth, which might be directly or indirectly associated with the immune function of colon. Thus, it was not surprising that immune function (802 genes) was identified with the core transcriptome of the colon, accounting for 7.03% of the total core gene expressions. Immune function in other compartments of the intestine of neonatal calves (at birth, 7, 21 and 42 days of life), such as the ileum and jejunum, has also been identified by Liang et al. [32] via RNA-seq based transcriptome analysis. However, of the 2499 immune related genes obtained from ImmPort (gene list shown in Additional file 1), our study only found 802 genes expressed in the colon, which may reflect tissue-specific gene expression patterns and demonstrate that the immune system of the colon was still immature (with 67.9% of the immune related genes being not expressed) at 2 days after birth. Such finding may provide molecular evidence that newborn calves are susceptible to gastrointestinal pathogenic infections [33]. In fact, it has been proposed that the susceptibility of newborns to pathogens is not from any inherent inability to mount an immune response, but rather, is caused by the fact that their immune system is unprimed [34]. The identification of the genes expressed in the mucosal immune system may also provide a molecular basis to suggest that the neonatal intestinal mucosal immune system is undergoing rapid changes during the early postnatal developmental period [32, 35].

In this study, very few differences were detected in the expression levels of the overall transcriptome and immune related genes of the colon based on varying colostrum feeding strategies. Colostrum contains a variety of bioactive components such as growth factors and hormones [9]. The lack of localized beneficial effects in TRT0 calves suggested that colostrum feeding time within 12 h after birth may result in no difference for the colonic development since the colon of calves was primed the same amount of bioactive components within 51 h of lifetime prior to the euthanasia. Moreover, passive transfer of IgG is generally considered successful when serum IgG concentrations in dairy calves are 10 g/L or greater between 24 and 48 h of age [36]. In the current study, although serum IgG at 48 h after birth in TRT6 (14.8 g/L) or TRT12 (15.3 g/L) calves was less than that in TRT0 (19.0 g/L), all of the calves had a serum IgG greater than 10 g/L and achieved adequate passive transfer following the administration of colostrum [16]. It was likely that all the calves had the capability to prevent challenges from pathogens through adequate passive transfer. Therefore, the higher blood IgG concentration – well above the threshold of successful passive transfer (i.e., 10 g/L) – observed in TRT0 calves may not result in effects for the colonic mucosal immune system development in calves at 51-h of age. Previous studies reported that the serum IgG concentration in dairy calves reached a maximum at 24 h of age and declined thereafter [37–39]. Since calves rely on colostral IgG for protection from pathogens until 3 or 4 weeks of age, when their own active immune system is functional [38], there would be a period before week 3 or 4 of age where blood IgG would be under the threshold of 10 g/L. Therefore, we speculate that the TRT0 calves may have greater IgG levels than TRT6 and TRT12 late in life when IgG concentrations decrease, such as later in week 3 or week 4. Ultimately, this needs further investigation**.**

In addition, it is worth noting that the colon transcriptome varies among calves even when under the same dietary treatment and within similar dam parity. While there was no overall change in the expression level of the selected genes, some individuals exhibited substantial changes (∆CPM, a difference with the overall average), with both positive and negative responses (∆CPM) occurring depending on selected genes (Additional file 7). Such large individual variation may lead to the lack of difference in the expression of immune related genes in the colon among different colostrum feeding strategies. Thus, there is a need to increase the amount of animals required in order to minimize the effects occurring from the animal itself and understand the actual impact of colostrum feeding time on the transcripotome profiles. In fact, variations among individual animals fed the same diet are commonly reported [40, 41]. When feeding the same diet, the reasons behind animal variation are likely related to animal behavior, physiology, genetics, dam condition, and microbial community [40–43]. In the current study, when considering the parity of the dam on calf transcriptome profile, expression of genes *RGS2* and *CCL14* were different between calves from primiparous and multiparous cows within TRT0 or TRT6. The mechanisms behind how the expression of *RGS2* or *CCL14* were altered by parity are unclear. The inconsistency across treatments for changes in gene expression due to parity (i.e., altered *RGS2* within TRT0, altered *CCL14* within TRT6, and no change within TRT12) indicates that the comprehensive genetic background of the dam, including the parity, may come into play.

Increasing evidence has shown that early colonized microbiota in the gastrointestinal tract play a crucial role in the development of the mucosal immune system and influence newborn health [44, 45]. In this study, a network-based analysis WGCNA, which combined the microbial abundance and transcriptome data in the colon with the same animal, confirmed that the immune related genes in the colon were associated with bacterial colonization, in particular with the abundance of *Lactobacillus* and *F. prausnitzii*, which were consistent with previous studies [32, 46]. In these studies, negative correlations between expression of mucosal TLRs and mucosa-attached bacteria were observed in young dairy calves. Moreover, it has been reported that *F. prausnitzii* displayed a negative association with calf diarrhea incidences [47] and the administration of *Lactobacillus* to newborn calves during the first week of life decreased diarrhea incidences [48]. Such findings suggest that a high abundance of *F. prausnitzii* or *Lactobacillus* during early life may play a vital role in maintaining colonic homeostasis and decreasing susceptibility to enteric infections in neonatal calves.

## Conclusion

Our results showed that there were few changes on transcriptome profiles of the colon in 51-h age of calves fed the first colostrum meal at different time within 12 h after birth, suggesting that transcriptome profiles of the colon in neonatal calves are independent of the timing of the initial colostrum meal within 12 h after birth. However, it is noted that there was a large individual variation in the transcriptomic profiles among the neonatal calves. The potential for improving calf mucosal immune system development through feeding colostrum meals immediately after birth still needs to be defined using a larger number of animals or a prolonged sampling time. Regardless, the transcriptome analysis provides a molecular understanding of the colonic biological function in neonatal calves and gene co-expression analysis extends knowledge on how gut tissue can interact with its luminal bacterial community, which may help to develop future nutritional management strategies for calf performance.

## Additional files

Additional file 1: List of immune related genes in the colon of 51-h age of dairy calves and the gene list obtained from ImmPort. (XLSX 12419 kb)

Additional file 2: Sample information details and sequencing results mapped to reference genome by Tophat2. (XLSX 10 kb)

Additional file 3: List of core genes in the colon. (XLSX 785 kb)

Additional file 4: Clustering dendrogram of genes showing module membership in colours. (TIFF 946 kb)

Additional file 5: WGCNA identification of colonic gene modules correlated with the bacterial abundance. The module-trait relationships indicates the correlation coefficients and *p*-values (in the brackets). The color scale bar shown in the right represents the Pearson correlation ranging from − 1 (green) to 1 (red). The bacterial population examined were *Lactobacillus* from the colon content (lac_C_P), *Lactobacillus* from the attached colon (lac_T_P), *Faecali bacterium prausnitzii* from the colon content (fea_C_P), *Faecali bacterium prausnitzii* from the attached colon (fea_T_P), *Bifidobacterium* from the colon content (bif_C_P), *Bifidobacterium* from the attached colon (bif_T_P), which were shown on the bottom. (PDF 8 kb)

Additional file 6: List of genes in significant modules identified using WGCNA. (XLSX 108 kb)

Additional file 7: Animal variations of 12 representative immune genes. (PDF 354 kb)

## Abbreviations

A2M: Alpha-2-macroglobulin
ACTG: Actin gamma
AHNAK: AHNAK nucleoprotein
BCR: Breakpoint cluster region
C: Multiparous cows
CALR: Calreticulin
CANX: Calnexin
CCL: Chemokine (C-C motif) ligand
CLDN: Claudin
CPM: Counts per million
DE gene: Differentially expressed gene
DES: Desmin
FC: Fold change
FDR: False discovery rate
GO: Gene ontology
H: Primiparous heifers
HSPA: Heat shock protein family A
Ig: Immunoglobulins
IL: Interleukin
LRP1: LDL receptor related protein
NFKB: Nuclear factor kappa B
PANTHER: Protein annotation through evolutionary relationship
PCA: Principal component analysis
PGLYRP: Peptidoglycan recognition protein
RGS: Regulator of G protein signaling
RNA-seq: RNA-sequencing
SCFA: Short chain fatty acid
SLC40A1: Solute carrier family 40 member 1
STAT: Signal transducers and activators of transcription
TCR: T cell receptor
TGF: Transforming growth factor
TLR: Toll-like receptors
TNF: Tumor necrosis factor
TPM: Tropomyosin
TRT0: Fed immediately after birth
TRT12: Fed at 12 h after birth
TRT6: Fed at 6 h after birth
WGCNA: Weighted gene co-expression network analysis
