# Supplementary figures and images for: Genome wide transcriptome analysis provides bases on colonic mucosal immune system development affected by colostrum feeding strategies in neonatal calves
Source: BMC Genomics. 2018 Aug 28;19:635. doi: 10.1186/s12864-018-5017-y (PMC6114731; doi:10.1186/s12864-018-5017-y)

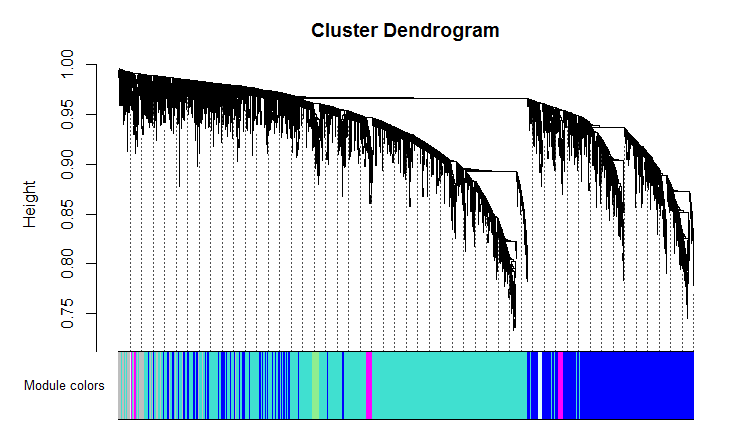

Supplement: Supplementary file 4 — Clustering dendrogram of genes showing module membership in colours. (TIFF 946 kb) [file 12864_2018_5017_MOESM4_ESM.tiff]

Module–trait relationships

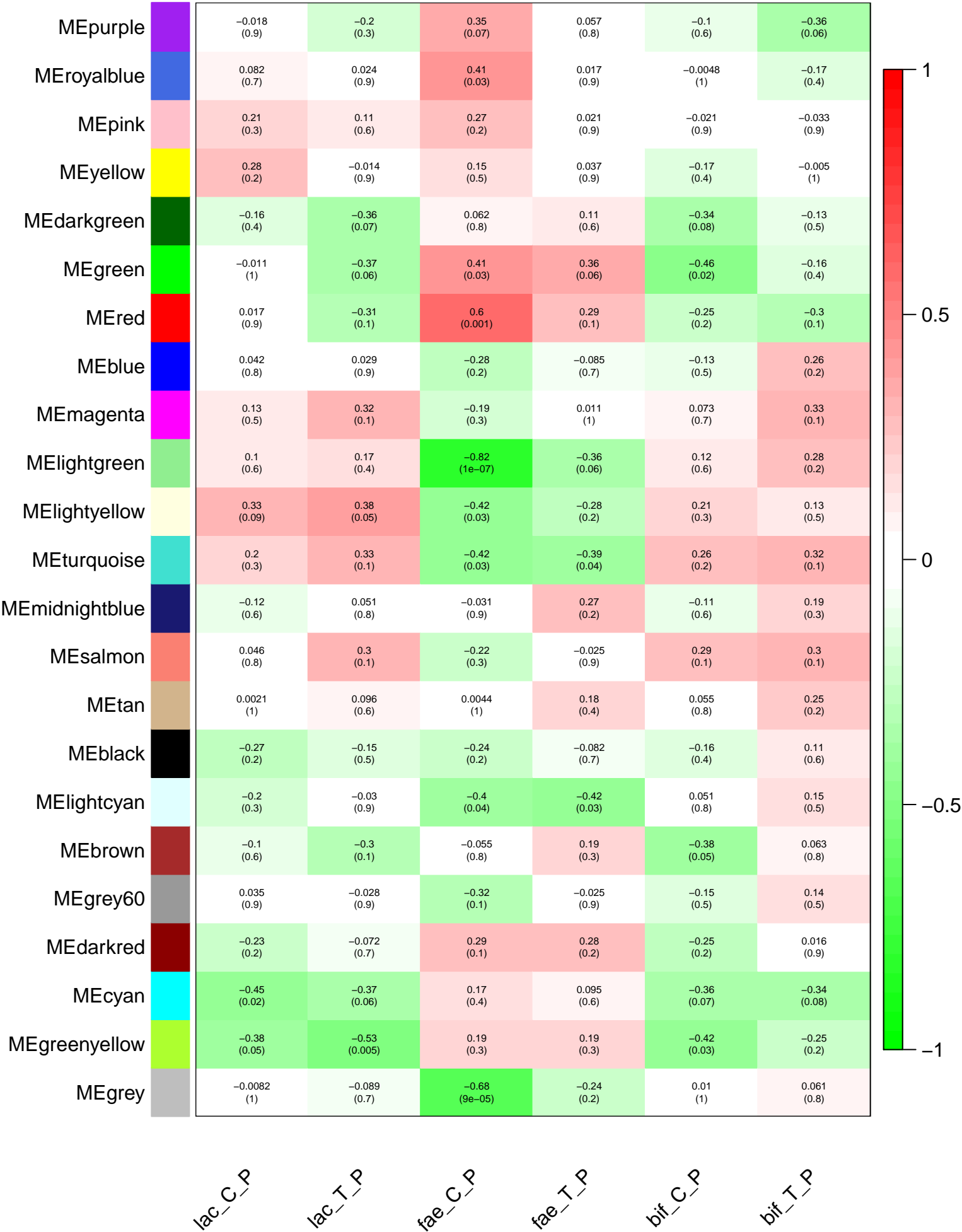

Supplement: Supplementary file 5 — WGCNA identification of colonic gene modules correlated with the bacterial abundance. The module-trait relationships indicates the correlation coefficients and p-values (in the brackets). The color scale bar shown in the right represents the Pearson correlation ranging from − 1 (green) to 1 (red). The bacterial population examined were Lactobacillus from the colon content (lac_C_P), Lactobacillus from the attached colon (lac_T_P), Faecali bacterium prausnitzii from the colon content (fea_C_P), Faecali bacterium prausnitzii from the attached colon (fea_T_P), Bifidobacterium from the colon content (bif_C_P), Bifidobacterium from the attached colon (bif_T_P), which were shown on the bottom. (PDF 8 kb) [file 12864_2018_5017_MOESM5_ESM.pdf]

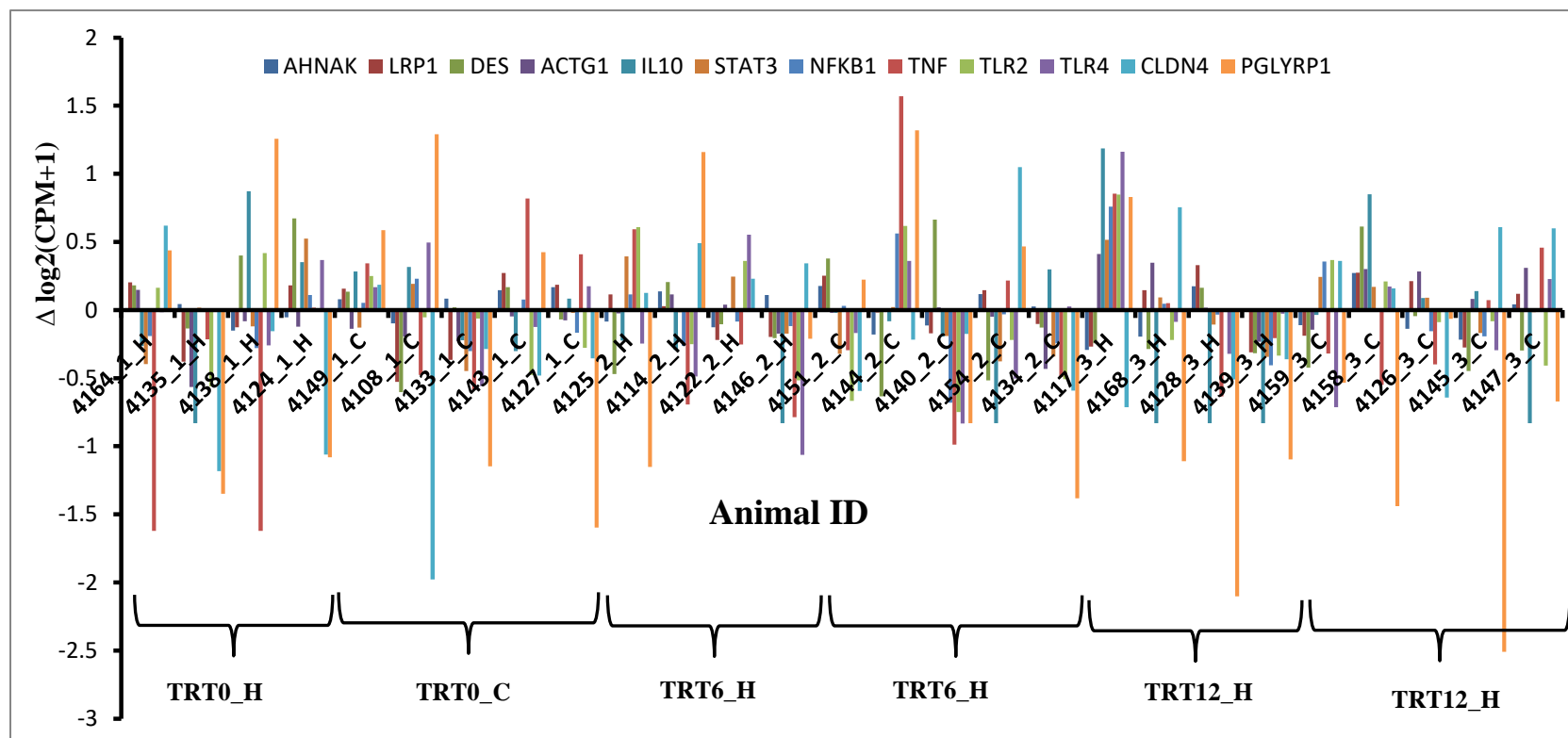

Supplement: Supplementary file 7 — Animal variations of 12 representative immune genes. (PDF 354 kb) [file 12864_2018_5017_MOESM7_ESM.pdf]
